# Bioinformatics-Guided Network Pharmacology Exploration of Taraxacum Officinale’s Renoprotective Effects Against Cisplatin-Induced Nephrotoxicity

**DOI:** 10.3390/nu17193092

**Published:** 2025-09-29

**Authors:** Ruiyi Hu, Shan Tang, Xufei Gao, Simin Qi, Shen Ren, Zi Wang, Xindian Li, Wei Li

**Affiliations:** 1 College of Chinese Medicinal Materials, Jilin Agricultural University, Changchun 130118, China; hry221510@163.com (R.H.); tangshan0423@163.com (S.T.); gxfgaoxufei@163.com (X.G.); rs0109@163.com (S.R.); wangzi8020@126.com (Z.W.); 2 National & Local Joint Engineering Research Center for Ginseng Breeding and Development, Changchun 130118, China; 3 School of Biomedical Sciences, Faculty of Medicine, The Chinese University of Hong Kong, Shatin, N.T., Hong Kong SAR, China; lena77_1998@outlook.com

**Keywords:** *Taraxacum officinale*, cisplatin, kidney injury, network pharmacology, GeneMANIA, PERK/eIF2α/ATF4

## Abstract

**Background/Objectives:** *Taraxacum officinale* F.H.Wigg. (Asteraceae), an edible plant and commonly used Chinese herbal medicine, has significant anti-inflammatory and antioxidant effects in the form of its root water extract (TRWE). Therefore, this study was designed to elucidate the principal pharmacological effects and underlying mechanisms of water extract from Taraxacum roots (TRWE) against cisplatin-induced nephrotoxicity through an integrated approach combining network pharmacology and experimental validation. **Methods:** Mechanistic prediction was performed using network pharmacology, molecular docking, and GeneMANIA-based functional analysis, followed by experimental validation via H&E staining, TUNEL, biochemical assays (blood urea nitrogen, BUN; creatinine, CRE; malondialdehyde, MDA; superoxide dismutase, SOD; and catalase, CAT), and Western blotting. **Results:** Network pharmacology identified 52 kidney injury-associated targets of *Taraxacum*. Functional enrichment analysis indicated their roles in apoptosis and endoplasmic reticulum stress, particularly through the PERK-mediated UPR pathway, suggesting the PERK/eIF2α/ATF4 axis as a potential key regulatory node. Animal experiments suggested that 100, 200, and 400 mg/kg inhibited cisplatin-induced increases in BUN, CRE, and MDA; restored SOD/CAT levels; and alleviated kidney apoptosis and endoplasmic reticulum stress via the PERK/eIF2α/ATF4 pathway. Molecular docking suggested strong binding of phytochemicals (caftaric acid, CTA; chlorogenic acid, CGA; caffeic acid, CA; and cichoric acid, CCA) to PERK, eIF2α, and ATF4. **Conclusions:** This study predicts that the PERK/eIF2α/ATF4 signaling pathway may be a critical mediator of TRWE’s potential renoprotective effects against cisplatin-induced acute kidney injury, offering a potential theoretical basis for further mechanistic exploration.

## 1. Introduction

Acute kidney injury (AKI), a prevalent renal disorder associated with reactive oxygen species (ROS), is characterized by elevated serum creatinine levels or reduced urine output. Cisplatin (CP) is a commonly used non-specific anticancer drug for the cell cycle in clinical practice [1]. It accumulates preferentially in the renal proximal tubules, with the mitochondria of proximal tubular epithelial cells being the primary intracellular site of accumulation. CP disrupts multiple mitochondrial functions, including mitochondrial membrane potential, the electron transport chain, the tricarboxylic acid (TCA) cycle, and oxidative phosphorylation. Furthermore, although the primary mechanism of cisplatin action involves binding to DNA, thereby inhibiting DNA replication and impairing cancer cell survival, it may also damage mitochondrial DNA replication within renal cells [2]. This process contributes to mitochondrial dysfunction and ultimately leads to renal impairment. The drug accumulates in the kidney, resulting in inflammation, injury, and cell death. According to reports, about 19% of deaths due to AKI are directly related to CP [3]. Notably, CP-induced nephrotoxicity coexists with ototoxicity [4], neurotoxicity [5]. and cardiotoxicity [6]. Mechanistically, the endoplasmic reticulum (ER) regulates protein biosynthesis and cellular homeostasis [7]. Endoplasmic reticulum stress (ERS)-associated apoptosis induced by CP stems from misfolded protein accumulation, which triggers ROS overproduction in the endoplasmic reticulum [8]. ERS-induced apoptosis involves multifactorial mechanisms, with the PERK/eIF2α/ATF4 axis serving as a critical pathway [9,10,11]. Current therapeutic strategies for CP-induced AKI offer only transient nephroprotection, and no approved clinical interventions are available that address the underlying molecular mechanisms [12,13,14]. Emerging evidence highlights nephroprotective phytochemicals from medicinal plants as promising candidates for mitigating CP-induced renal damage, warranting systematic exploration of their therapeutic mechanisms.

*Taraxacum officinale* F.H.Wigg., also known as dandelion, Asteraceae, or perennial herbs, is a medicinal plant recognized by the World Health Organization (WHO). Dandelion has been used as a traditional medicine since the 16th century. German botanist Leonhard Fuchs introduced the utilization of dandelion in the treatment of blisters, diarrhea, and spleen and liver diseases [15]. With the continuous development of research, dandelion also has diuretic [16], anti-tumor [17], anti-oxidant, and anti-inflammatory activities [18]. Phytochemical studies have shown that *Taraxacum* possesses a diverse range of phytochemicals, including phenolics, flavonoids, terpenes, and coumarins. Of these bioactive constituents, phenolics are not only the most abundant compounds but also considered the primary active compounds in *Taraxacum* [19]. Recently, *Taraxacum* has been found to potentially decrease susceptibility to various diseases, including tumors [17]. Traditional use also suggests potential benefits for renal health, though its specific role in enhancing antioxidant and antiproliferative effects in the kidney requires further validation [20]. However, scanty data have been reported in relation to the protective effects of *Taraxacum* against CP-induced nephrotoxicity, whose mechanism of action needs to be further studied.

Network pharmacology (NP) provides a comprehensive framework for analyzing complex diseases by deciphering drug-target interactions and associated biological pathways [21,22]. Computational methods such as molecular docking enable detailed investigation of binding conformations and molecular stability in protein–drug systems. Our study integrated NP strategies with molecular docking to elucidate the potential mechanisms underlying *Taraxacum officinale* in treating renal injury. Furthermore, GMFA (GeneMANIA-based Functional Association) analysis enhanced NP predictions by investigating functional relationships among hub targets. Consequently, this study evaluated the effects of the aqueous extract of *Taraxacum officinale* roots (TRWE) on CP-induced renal damage using NP and an AKI murine model. Additional validation focused on TRWE’s modulation of PERK/eIF2α/ATF4 signaling pathway proteins, caspase family proteins, and renal injury-related biomarkers. The results demonstrate that TRWE may mitigate nephrotoxicity by potentially suppressing oxidative stress and ERS, which could reduce apoptotic activity.

## 2. Materials and Methods

### 2.1. Chemicals and Reagents

*Taraxacum officinale* F.H.Wigg. was collected in Jingyu, Jilin (May 2022), desiccated to constant weight, and cryopreserved (−20 °C). Voucher specimen 20220510001 is archived in the Chinese Medicinal Materials Botanical Garden.

CP (≥99% purity) was from Sigma Chemicals (St. Louis, MO, USA). Assay kits for BUN, CRE, MDA, SOD, and CAT were from Nanjing Jiancheng Biological Research Institute (Nanjing, China). BCA protein concentration assay kits and hematoxylin and eosin (H&E) were provided by Beyotime Co., Ltd. (Shanghai, China). Immunofluorescence staining was obtained from BOSTER Biological Technology (Wuhan, China), and the TUNEL kits were obtained from Roche Applied Science (Shanghai, China). Antibodies for Bax, Bad, Bcl-2, Bcl-xL, cytochrome c, caspase 3, cleaved caspase 3, p-PERK, PERK, p-eIF2α, eIF2α, ATF4, CHOP, caspase 12, cleaved caspase 12, and Gapdh were obtained from Cell Signaling Technology (Danvers, MA, USA). HPLC-grade methanol was obtained from Fisher Chemicals (Waltham, MA, USA).

### 2.2. Preparation of TRWE and HPLC Analysis

Dried *Taraxacum* powder (5 g) underwent aqueous extraction (50 mL) via 37 °C sonication for 1 h. HPLC analysis used 20 µL supernatant. Methanol-dissolved standards (CTA 0.0402, CGA 0.0404, CA 0.020, and CCA 0.014 mg/mL) were similarly analyzed with 20 µL injections.

**Figure 1:**
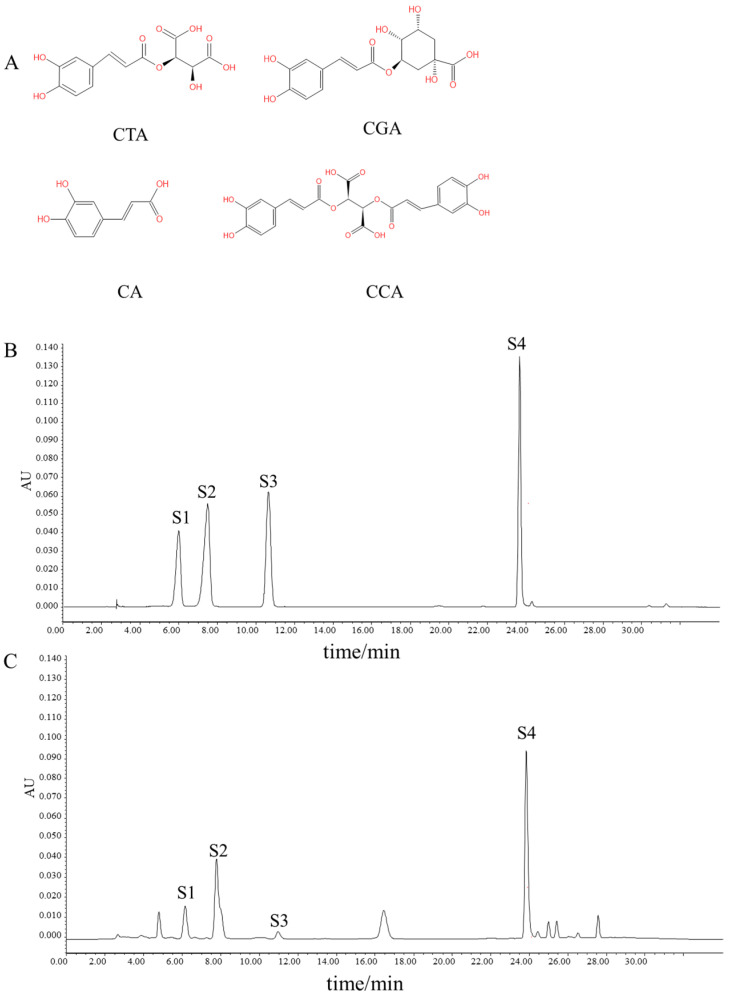
HPLC chromatograms of phenolic compounds in Taraxacum. (**A**) The structural formula of four phenolic acids. (**B**) Reference standard. (**C**) Sample. S1–S4 correspond to CTA, CGA, CA, and CCA, respectively.

Quantification of *Taraxacum* constituents (CTA/CGA/CA/CCA) employed Waters e2695 HPLC with UV detection [23]. Separation used a Hypersil BDS2 column (250 × 4.6 mm, 5 μm) with 0.2% aqueous phosphoric acid (A) and methanol (B) gradient as follows: 25% B (0–15 min), 25–50% B (15–25), 50% B (25–28), 50–25% B (28–29), and 25% B (29–34). Separation was performed at 1.0 mL/min and 35 °C with detection at 330 nm. The resultant chromatograms are shown in Figure 1B,C.

### 2.3. Acquisition of the Targets of Kidney Injury and Phenolic Compounds in Taraxacum

Phenolic compounds from *Taraxacum* HPLC analysis were queried in PubChem to generate SDF files, subsequently processed through SwissTargetPrediction (http://www.swisstargetprediction.ch/, accessed on 5 April 2022) [24,25]. Targets with probability ≥0 were retrieved for “Homo sapiens”. UniProt IDs were converted to gene symbols using Retrieve/ID mapping in the UniProt database.

Therapeutic targets for kidney injury were identified through integrated analysis of GEO (GSE227970; 3 normal vs. 3 injury samples; https://www.ncbi.nlm.nih.gov/geo/, accessed on 5 April 2022), GeneCards (https://www.genecards.org, accessed on 5 April 2022), TTD (http://db.idrblab.net/ttd/, accessed on 5 April 2022), PharmGKB (https://www.pharmgkb.org/, accessed on 5 April 2022), OMIM (https://www.omim.org/, accessed on 5 April 2022), and NCBI (https://www.ncbi.nlm.nih.gov/gene, accessed on 5 April 2022 ) [26]. AKI-associated differentially expressed genes (DEGs) were screened using GEO2R (|logFC| > 2, *p* < 0.05). Consolidated targets from database intersections were cross-referenced with *Taraxacum* phenolic compound targets through bioinformatic integration.

### 2.4. Network Construction and Enrichment Analysis

The *Taraxacum* phenolic–kidney injury network was visualized via Cytoscape 3.9.0. PPI networks were generated using STRING (https://string-db.org, accessed on 5 April 2022), with TSV data subjected to CytoHubba topological analysis (Degree algorithm) in Cytoscape to identify key therapeutic targets [27].

Bioinformatic analyses of *Taraxacum*-related kidney injury targets were performed using R 4.1.2. Gene Ontology (GO) and KEGG pathway enrichment analyses were conducted, with results visualized through bar plots and categorical diagrams generated via the Weishengxin Cloud Platform (https://www.bioinformatics.com.cn, accessed on 23 December 2024) and Metware Biotechnology (https://www.metwarebio.com, accessed on 23 December 2024).

We developed the GeneMANIA-based Functional Association (GMFA) framework within GeneMANIA to identify hub target-associated genes, systematically prioritizing 20 functional interactors per hub through gene–gene network association strength. Biological relevance was annotated via GO enrichment and KEGG pathway mapping [28].

### 2.5. Molecular Docking

Crystal structures of ATF4 (PDB ID: 1CI6), eIF2α (PDB ID: 1KL9), and PERK (PDB ID: 4X7K) were acquired from the Protein Data Bank (https://www.rcsb.org, accessed on 8 April 2022). Phenolic compound configurations were generated with ChemDraw (ChemDraw 21.0.0, PerkinElmer, Waltham, MA, USA) and 3D (Chem3D 21.0.0, PerkinElmer, USA) structural optimization using MM2 energy minimization in ChemBio3D Ultra 14.0.0.117. Protein preparation involved dehydration and ligand removal via PyMOL 2.4.1 (Schrödinger, LLC, New York, NY, USA). Hydrogenation and charge calculations were performed using AutoDockTools-1.5.6 (Scripps Research Institute, La Jolla, CA, USA), subsequently converting ligands and receptors to pdbqt format. Molecular docking was executed through AutoDock Vina 1.2.2 (Scripps Research Institute, USA) with optimized grid parameters [29]. Binding conformations were visualized using BIOVIA Discovery Studio 2019 (Dassault Systèmes, Velizy-Villacoublay, France) [30].

### 2.6. Animals and Experimental Design

Eight-week-old male ICR mice (weighing 22–25 g) were procured from Changchun YISI Experimental Animal Co., Ltd. with Certificate of Quality No. SCXK (JI)-2022-0003 (Changchun, China). The mice were housed in plastic cages and given free access to food and water under controlled conditions of temperature (23.0 ± 2.0 °C), humidity (60 ± 10%), and a 12 h light/dark cycle. All animal experiments strictly complied with the Guide for Ethical Committee of Laboratory Animals in Jilin Agricultural University (Approval number: 20220802003). There is no positive drug for the treatment of kidney injury caused by CP [31]. After one week of adaptive feeding, mice were randomly allocated into five groups (*n* = 8 per group) using a computer-generated random number sequence (randomization seed: 20220809). Allocation was stratified by body weight to ensure balanced group assignment. To minimize confounding effects, cage positions were rotated daily, and all experimental procedures (e.g., drug administration and sample collection) were performed in randomized order by an operator blinded to group identities. The mice in the CP+TRWE groups received intragastric administration of TRWE for ten consecutive days, while the normal group and the CP group were administered 0.9% normal saline. All mice underwent fasting for a minimum period of 12 h. On the 7th day of treatment, acute kidney injury was induced by intraperitoneal injection of CP (20 mg/kg) [32,33], except for the normal group. Then, a continuous administration of the treatments was performed for three consecutive days, followed by dissection on the 10th day. Mice were allowed free water access during experiments. After dissection, the kidneys were collected and weighed, and the kidney index (%) was calculated as (kidney weight/body weight) × 100%. The renal tissues were then divided into portions for morphological analysis (fixed in 10% formalin, mass/volume ratio) and rapidly frozen in aluminum foil at −80 °C for homogenate preparation and immunoblotting. All experimental procedures and animal handling were performed in accordance with the guidelines of the Institutional Animal Care and Use Committee.

### 2.7. Assessment of Biochemical Parameters and H&E Staining

Renal CAT, SOD, MDA, and serum BUN/CRE levels were quantified per manufacturer protocols [34,35,36]. Tissue homogenates (1:9 *w*/*v* in saline, ice bath) underwent dual centrifugation (10 min, 300× *g*, 4 °C) prior to analysis.

H&E staining was conducted following the method described earlier [37,38]. Renal tissues were fixed in 4% paraformaldehyde (24 h), paraffin-embedded, sectioned (5 μm), and stained with H&E for histological examination using a Leica TCS SP8 microscope (Solms, Germany).

### 2.8. Apoptotic Cell Assay and Immunofluorescence (IF) Staining

The TUNEL staining procedure was conducted according to the previously established protocol [39,40]. The extent of apoptosis was evaluated using TUNEL apoptosis detection kits. Finally, a Leica microscope (Leica TCS SP8, Solms, Germany) was utilized for observing the nucleus expression of TUNEL-positive cells.

IF staining was conducted as described in previous reports [39,41]. Briefly, sections were incubated overnight with CHOP/ATF4 antibodies (1:200, 4 °C), washed with PBS (3×), and then incubated with SABC-DyLight448 secondary antibody (BOSTER, Wuhan, China; 37 °C, 30 min). Nuclear counterstaining with DAPI preceded imaging under dark conditions (Leica TCS SP8, Solms, Germany), with IF intensity quantified via Image-Pro Plus 6.0 (Media Cybernetics, Rockville, MD, USA).

### 2.9. Western Blotting Analysis

Western blot analysis was conducted as described in previous studies [42,43,44]. Renal proteins were extracted using Trident RIPA Lysis Buffer (GeneTex, Irvine, CA, USA). Equal protein quantities underwent SDS-PAGE and were transferred to PVDF membranes. After blocking with 5% skim milk (2.5 h), membranes were washed thrice with TBS-T. Primary antibodies targeting Bad (1:1000), Bcl-xL (1:1000), cytochrome c (1:1000), Bax (1:1000), Bcl-2 (1:1000), caspase 3/cleaved caspase 3 (1:1000), p-PERK/PERK (1:2000), p-eIF2α/eIF2α (1:2000), ATF4 (1:1000), CHOP (1:1000), caspase 12/cleaved caspase 12 (1:1000), and Gapdh (1:4000) were incubated overnight at 4 °C. Following secondary antibody (1:20,000) incubation (2 h), chemiluminescent signals were detected using the Tanon 5200 Multi system (Tanon Biotechnology, Shanghai, China). Band intensity quantification was performed with Quantity One 4.6.2 software (Bio-Rad Laboratories, Hercules, CA, USA).

### 2.10. Statistical Analysis

All data are presented as the mean ± standard deviation (mean ± S.D.) obtained from various experiments and were analyzed using one-way analysis of variance (ANOVA) followed by the Bonferroni post hoc test. Statistical graphs were generated using GraphPad Prism 8.0.2 software (GraphPad Software, Inc., San Diego, CA, USA). The statistically significant difference was considered as *p* < 0.05, *p* < 0.01, or *p* < 0.001.

## 3. Results

### 3.1. Network Pharmacology Revealed the Target Characteristics of Taraxacum for Kidney Injury Through GO Enrichment Analysis and KEGG Enrichment Analysis

**Figure 2:**
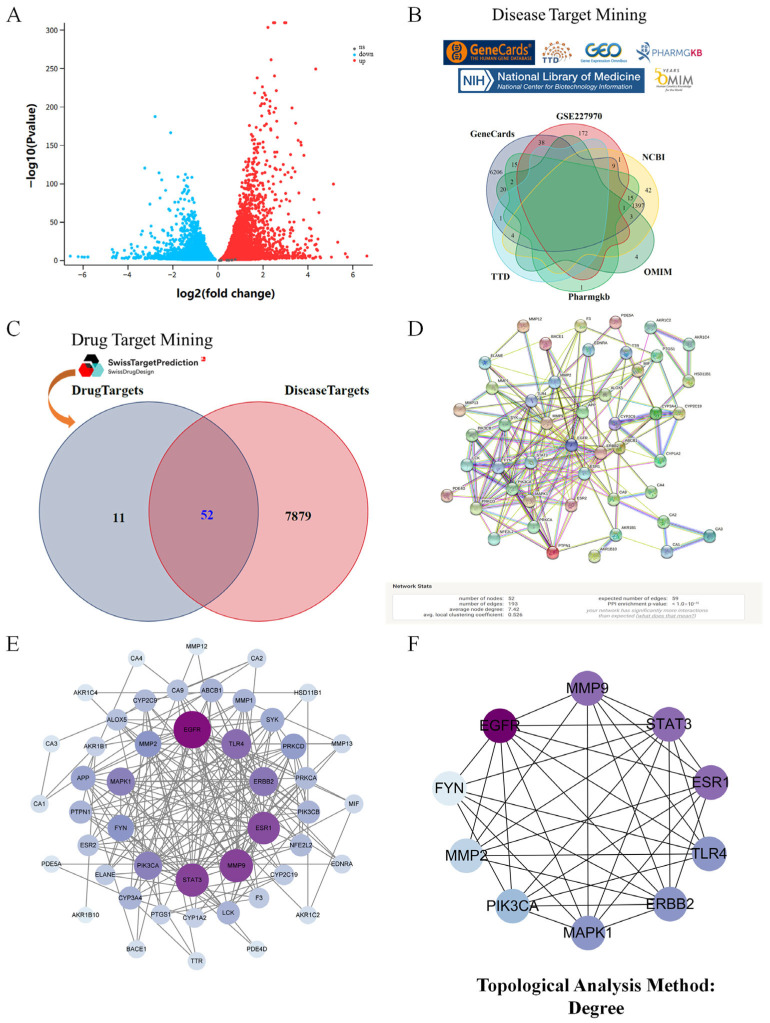
NP revealed the target characteristics of phenolic compounds in Taraxacum for kidney injury through bioinformatics analysis based on target genes. (**A**) The volcano map of differential genes. (**B**) Venn diagram of intersecting genes from disease databases. (**C**) Venn diagram of common targets of phenolic compounds in Taraxacum for kidney injury. (**D**) Construction and analysis of an interactive PPI network based on the STRING database, involving 52 Taraxacum phenolic compounds targeting kidney injury. (**E**) The protein–protein interaction (PPI) network based on targets of phenolic compounds in Taraxacum on kidney injury. Nodes represent different proteins. Edges represent protein–protein associations; the size of the circle indicates the strength of data support. (**F**) The PPI network using the CytoHubba plugin in Cytoscape.

Phenolic acid quantification using an external standard method quantified CTA (0.083 mg/g), CGA (0.097 mg/g), CA (0.505 mg/g), and CCA (0.069 mg/g) in dried samples (Figure 1A). NP identified 63 phenolic compound targets and 7931 kidney injury-related targets from multi-database integration (Figure 2B, Table 1 and Table S1). Transcriptomic analysis of GES66360 revealed 580 AKI-associated DEGs (Figure 2A). Intersection analysis identified 52 overlapping targets (Figure 2C, Table S2). A PPI network was constructed (Figure 2D), with EGFR, MMP9, STAT3, ESR1, TLR4, ERBB2, MAPK1, PIK3CA, MMP2, and FYN ranked as the top 10 key genes (Figure 2E). The CytoHubba plugin further prioritized these core targets linked to renal protection, with biological functions systematically evaluated via degree centrality (Figure 2F).

**Figure 3:**
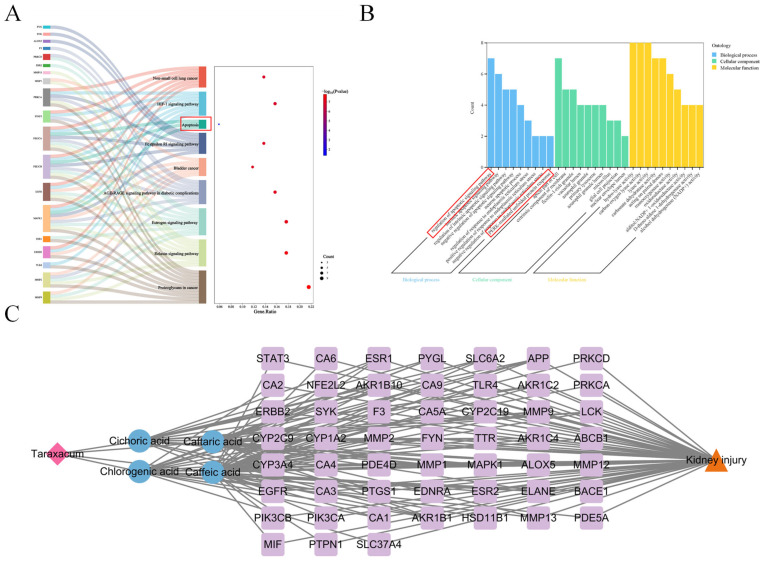
GO and KEGG enrichment analysis, the compound–target network of phenolic compounds in Taraxacum targeting kidney injury. (**A**) Sankey diagram of KEGG enrichment analysis for 9 signaling pathways involved in Taraxacum against kidney injury (Red box: Area of interest). (**B**) GO enrichment analysis revealing the BP, CC, and MF functional attributes of Taraxacum against kidney injury (Red box: Area of interest). (**C**) The compound–target network illustrating the interactions between phenolic compounds in Taraxacum and their targets.

Enrichment analysis systematically reveals biological processes linked to core targets by identifying gene/protein-associated functions. In this study, Gene Ontology (GO) analysis of 52 phenolic compound targets suggested their potential involvement in apoptotic signaling regulation, the PERK-mediated unfolded protein response, and endoplasmic reticulum stress modulation (Figure 3B, Table S3). Parallel KEGG pathway enrichment analysis suggested potential associations with the AGE-RAGE signaling pathway in diabetic complications, apoptosis, and the HIF-1 signaling pathway (Figure 3A). A Cytoscape-based multidimensional network model was established to interconnect *Taraxacum*-derived components, phenolic acids, therapeutic targets, and renal injury mechanisms (Figure 3C).

### 3.2. Functional Network Analysis of Top 10 Hub Targets Underlying Taraxacum’s Anti-Kidney Injury Effects Using GeneMANIA

**Figure 4:**
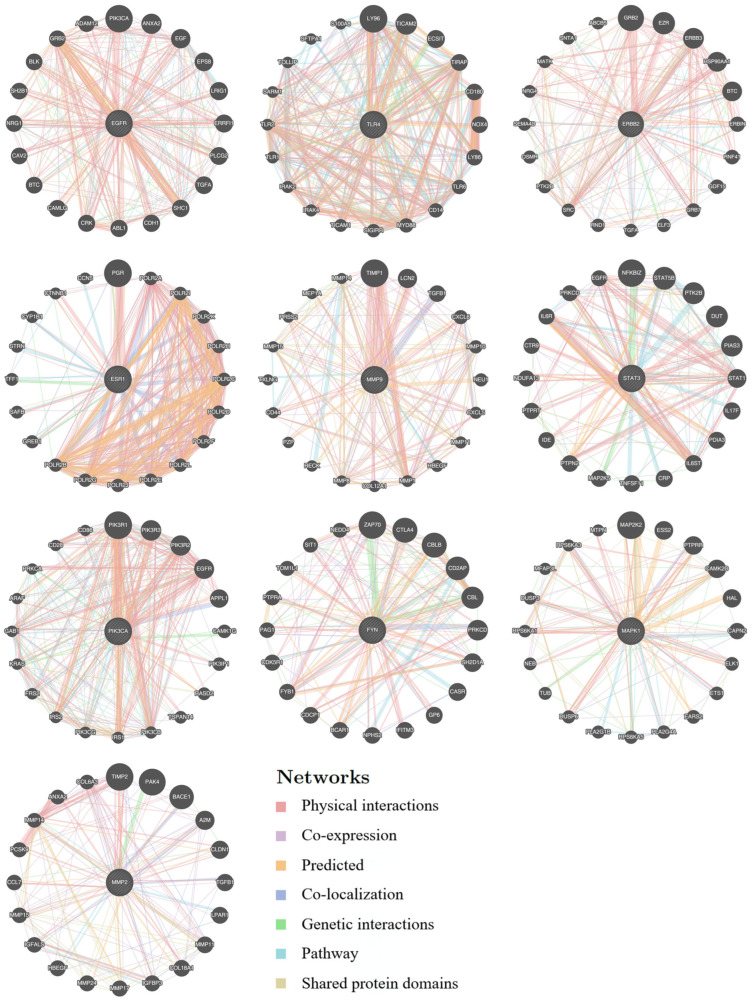
GeneMANIA Functional Association (GMFA) network analysis revealed functionally related genes associated with the top 10 hub targets of *Taraxacum* and established an extended potential target database (GMFA-ED) for kidney injury.

**Figure 5:**
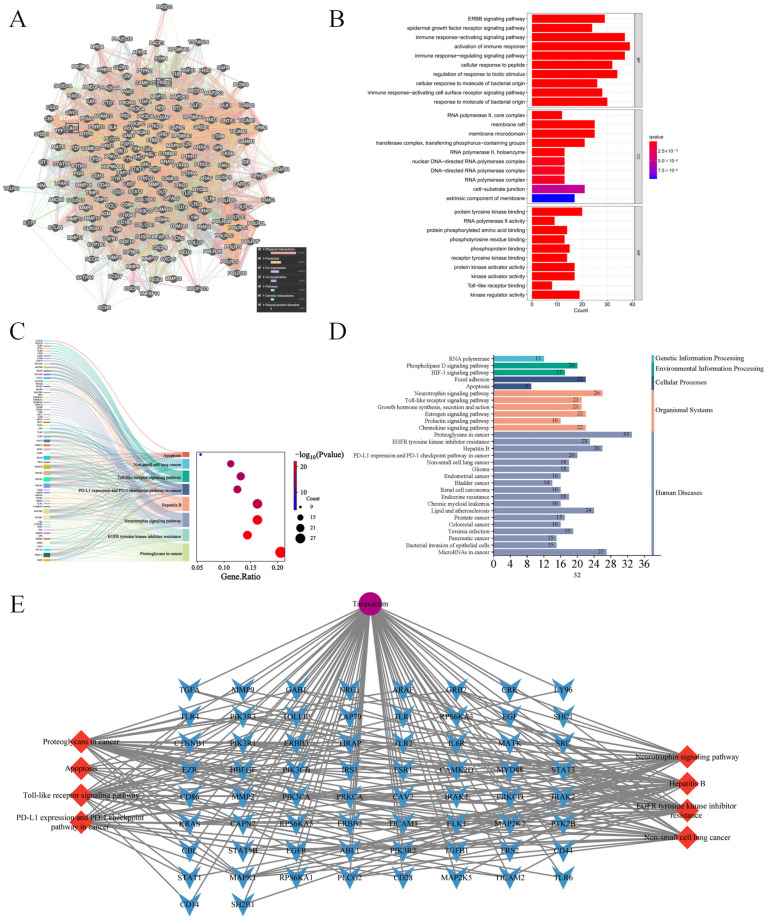
GO and KEGG enrichment analysis and the target–pathway network of *Taraxacum* targets identified in the GMFA-ED dataset. (**A**) The FGN of all 196 genes identified as new potential targets for *Taraxacum* against kidney injury. (**B**) Bar plots of the top 10 GO-BP, GO-CC, and GO-MF terms for enriched targets. (**C**) Sankey diagram of KEGG enrichment analysis for 8 signaling pathways involved in *Taraxacum* against kidney injury. (**D**) Classification diagram of pathway enrichment results for *Taraxacum* against kidney injury. (**E**) Target–pathway network of interactions between *Taraxacum* targets against kidney injury and predicted targets based on GMFA.

This study analyzed 10 core genes from GeneMANIA. A functional genomic expansion database (GMFA-ED) for *Taraxacum’s* nephroprotection was constructed by integrating 20 associated genes, ultimately identifying 196 key genes post-deduplication (Figure 4, Table S4). A functional gene network (FGN) was developed using GeneMANIA with multi-dimensional parameters (co-expression and interactions) and mapping disease-associated gene relationships (Figure 5A). GO analyses via R identified 1366 biological process (BP), 84 cellular component (CC), and 152 molecular function (MF) enriched terms (*p* < 0.05; Figure 5B–E, Table S5). GMFA-ED revealed multi-target synergies involving immune homeostasis, transmembrane signaling, and epigenetic regulation. KEGG enrichment analysis suggested three potential regulatory mechanisms in renal apoptosis: (1) activation of the HIF-1 pathway via HIF-1α stabilization may inhibit apoptosis under hypoxia; (2) suppression of TLR4/NF-κB signaling could reduce expression of TNF-α and caspase-3; (3) EGFR activation might promote cell survival through the PI3K/AKT and MAPK/ERK pathways. This integrated genomic database and network analyses systematically clarify *Taraxacum*’s multi-target renoprotective mechanisms. Based on these findings and the established role of CP in inducing ERS, we subsequently conducted molecular docking analyses and in vivo experiments to investigate whether TRWE modulates key components of the unfolded protein response signaling pathway, specifically examining its effects on the PERK-eIF2α-ATF4 signaling axis.

### 3.3. Molecular Docking of Active Compounds with Target Proteins

**Figure 6:**
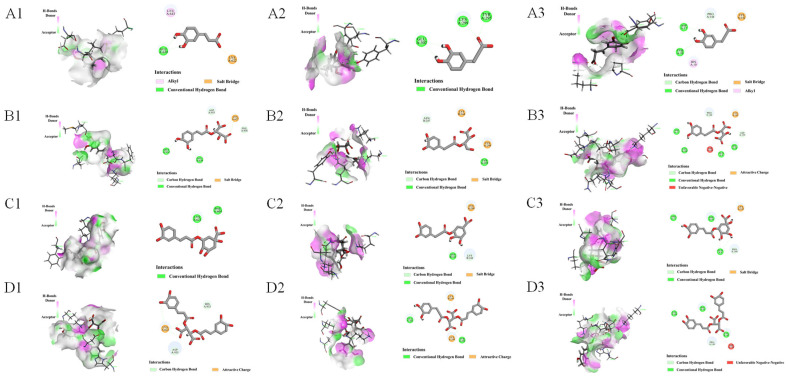
Molecular docking. (**A1**,**B1**,**C1**,**D1**) Action of CA, CTA, CGA, and CCA on PERK, respectively. (**A2**,**B2**,**C2**,**D2**) Action of CA, CTA, CGA, and CCA on ATF4, respectively. (**A3**,**B3**,**C3**,**D3**) Action of CA, CTA, CGA, and CCA on eIF2α, respectively.

Firstly, four phenolic compounds were combined with ATF4, eIF2α, and PERK, respectively, to verify the interaction between phenolic acids and target proteins. It is commonly accepted that a lower binding capacity results in a more stable binding between the ligand and the receptor. Generally, a docking score ≤ −5 kcal/mol suggests a stable interaction between the two docked entities. Table S6 shows that the docking fractions were less than −5 kcal/mol, which can verify the binding ability between components and targets to some extent. The four phenolic acids formed hydrogen bonds and van der Waals forces with ATF4, eIF2α, and PERK through different amino acid residues (Figure 6), providing theoretical support for subsequent animal experiments.

### 3.4. TRWE Ameliorated CP-Induced Oxidative Stress Injury

**Figure 7:**
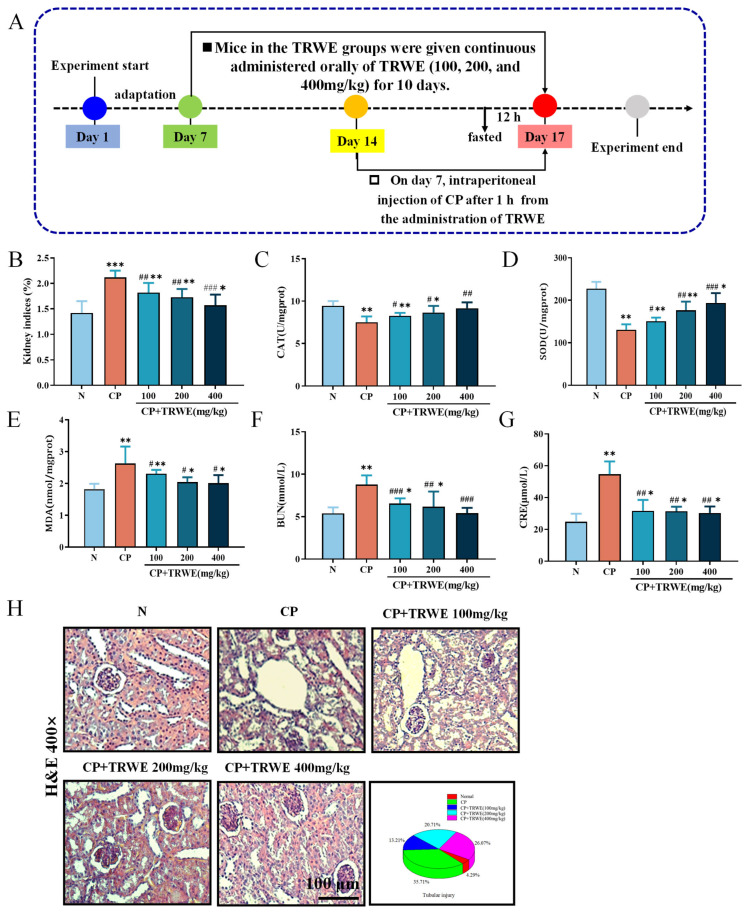
The protective effects of TRWE against CP-induced kidney injury. (**A**) Experimental design of the renoprotective effect of TRWE on mice. (**B**) Kidney indices. The kidney levels of (**C**) CAT, (**D**) SOD, and (**E**) MDA. CP increased serum (**F**) BUN and (**G**) CRE levels (*n* = 8 in each group). (**H**) Kidneys stained with H&E (400×) and the percentage of tubular injury (*n* = 3 in each group). All values are expressed as mean ± S.D. * *p* < 0.05, ** *p* < 0.01, *** *p* < 0.001 vs. normal group; ^#^ *p* < 0.05, ^##^ *p* < 0.01, ^###^ *p* < 0.001 vs. CP group.

Figure 7A shows the experimental design of cisplatin (CP)-induced kidney injury in mice. The health status of the mice was preliminarily assessed using organ indices. The kidney index was significantly higher in the CP group compared with the control group (*p* < 0.001). In contrast, TRWE treatment significantly reduced the kidney index in a dose-dependent manner (*p* < 0.05, *p* < 0.01, *p* < 0.001). Furthermore, oxidative stress markers, including CAT, SOD, and MDA, were measured. As shown in Figure 7C,D, CP exposure resulted in a significant decrease in CAT and SOD levels in kidney homogenates compared with the normal group (*p* < 0.05 or *p* < 0.001). However, supplementation of TRWE for 10 consecutive days ameliorated the reduction of kidney SOD and CAT content caused by CP injection (*p* < 0.05, *p* < 0.01, or *p* < 0.001). As the final product of lipid peroxidation, the content of MDA in the kidneys of CP-exposed mice increased significantly. However, pretreatment with TRWE clearly inhibited the observed increase (Figure 7E). Notably, treatment with TRWE (at doses of 100, 200, and 400 mg/kg) ameliorated the impaired renal function caused by CP-induced nephrotoxicity in a dose-dependent manner. Particularly, the TRWE group receiving high doses (400 mg/kg) exhibited kidney function levels that were almost restored to normal, which suggests a protective effect of TRWE on kidney tissues against CP-induced nephrotoxicity (*p* < 0.05, *p* < 0.01, or *p* < 0.001).

### 3.5. TRWE Reversed CP-Induced Kidney Dysfunction in Mice

BUN and CRE are often used as indicators of the degree of kidney tissue damage. As summarized in Figure 7F,G, compared to the normal group, CP injection at a dose of 20 mg/kg significantly elevated the serum CRE and BUN levels, while a significant decrease in these parameters was observed in the TRWE-treated groups (*p* < 0.01 or *p* < 0.001).

H&E staining (Figure 7H) demonstrated preserved glomerular architecture and tubular integrity with normal extracellular matrix distribution in controls. The CP group exhibited structural deterioration, including tubular dilation, epithelial necrosis, intratubular casts, nuclear pyknosis, vacuolar degeneration, and interstitial edema. TRWE pretreatment notably restored glomerular clarity, reduced tubular necrosis/cast formation, and normalized cellular morphology, with maximal efficacy at 400 mg/kg (*p* < 0.05 or *p* < 0.01). These findings suggest TRWE mitigates CP-induced renal pathology.

### 3.6. TRWE Inhibited CP-Induced Apoptosis in Kidney Tissues

**Figure 8:**
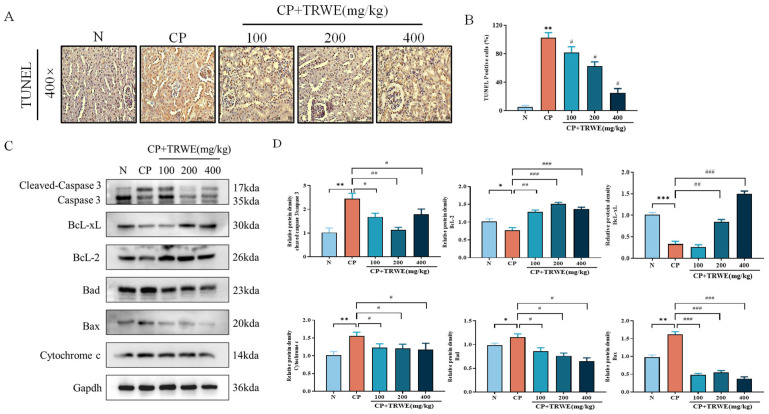
The protective effects of TRWE against CP-induced apoptosis. (**A**) Kidneys stained with TUNEL (400×). (**B**) The percentage of TUNEL-positive cells. (**C**) The protein expression levels of Bax, Bcl-2, Bad, Bcl-xL, cytochrome c, caspase 3, caspase 9, and cleaved caspase 3 measured by Western blotting with specific primary antibodies and standardized to that of Gapdh. (**D**) Quantification of relative protein expression analysis levels according to densitometric analysis. All values are expressed as mean ± S.D. (*n* = 3 in each group). * *p* < 0.05, ** *p* < 0.01, *** *p* < 0.001 vs. normal group; ^#^ *p* < 0.05, ^##^ *p* < 0.01, ^###^ *p* < 0.001 vs. CP group.

To assess the level of kidney apoptosis following CP exposure, TUNEL analysis was performed. As demonstrated in Figure 8A,B, TUNEL staining revealed intact nuclear morphology in controls, whereas CP exposure induced tubular epithelial nuclear hyperchromasia, widespread lysed nuclei, and elevated TUNEL-positive cells. TRWE (400 mg/kg) significantly attenuated these pathological markers.

To further elucidate the molecular mechanism underlying CP-induced apoptosis in kidney tissues, Western blotting (Figure 8C,D) was used to demonstrate CP-mediated upregulation of pro-apoptotic proteins (Bad, Bax, cytochrome c, and cleaved caspase-3) and suppression of anti-apoptotic factors (Bcl-2 and Bcl-xL). TRWE pretreatment upregulated the expression of Bcl-2 and Bcl-xL, while it downregulated Bad and inhibited cytochrome c release and caspase activation (*p* < 0.05, *p* < 0.01, or *p* < 0.001), corroborating its anti-apoptotic efficacy.

### 3.7. TRWE Reduced CP-Induced ERS Response

**Figure 9:**
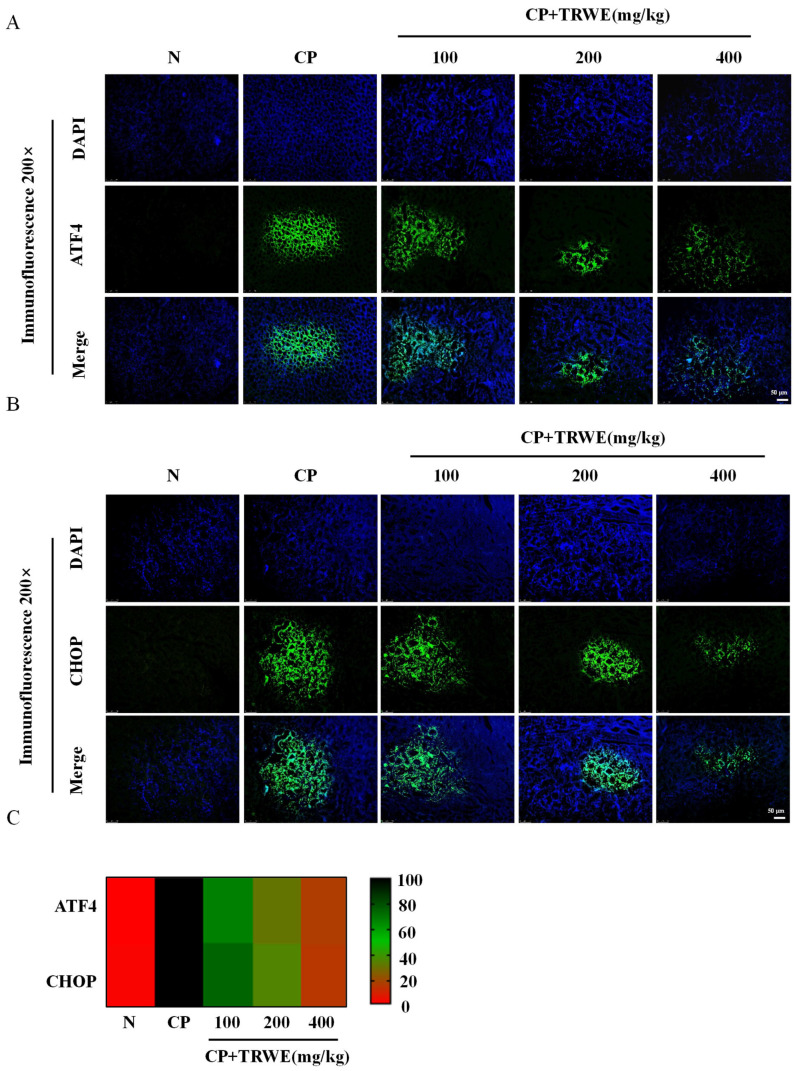
The protective effects of TRWE against CP-induced ERS. The expression level of (**A**) ATF4 and (**B**) CHOP in kidney tissue sections isolated from different groups as assessed by IF (200×). (**C**) Heat map showing analysis of the correlation of fluorescence intensity. All values are expressed as mean ± S.D. (*n* = 3 in each group).

For analyzing ERS in the kidney tissues after CP exposure, the IF staining method was used to detect the ERS-related proteins ATF4 and CHOP. Compared with the CP group, the levels of ATF4 and CHOP were significantly reduced in TRWE treatment groups. The findings illustrated in Figure 9A–C demonstrate that TRWE could exert kidney protection by inhibiting the ERS response (*p* < 0.05 or *p* < 0.01).

**Figure 10:**
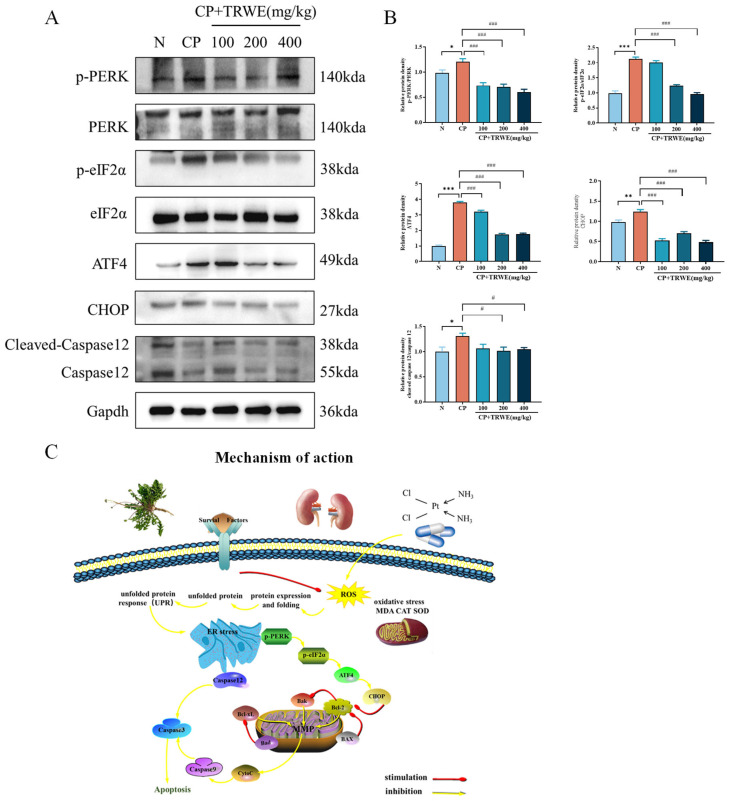
The protective effects of TRWE against CP-induced ERS by PERK/eIF2α/ATF4 signaling pathways. (**A**) The protein expression levels of p-PERK, PERK, p-eIF2α, eIF2α, ATF4, CHOP, caspase 12, and cleaved caspase 12 standardized to that of Gapdh. (**B**) Quantification of relative protein expressions. (**C**) Mechanism of action. All values are expressed as mean ± S.D. (*n* = 3 in each group). * *p* < 0.05, ** *p* < 0.01, *** *p* < 0.001 vs. normal group; ^#^ *p* < 0.05, ^###^ *p* < 0.001 vs. CP group.

To further investigate the impact of TRWE on the mechanism of ERS response, as depicted in Figure 10A,B, Western blotting analysis was used. The results showed that the protein expressions of p-PERK, PERK, p-eIF2α, eIF2α, ATF4, CHOP, and caspase 12 in the CP exposure group were significantly activated when compared with normal mice (*p* < 0.05, *p* < 0.01, or *p* < 0.001). On the contrary, administration of TRWE effectively significantly inhibits the protein expressions of p-PERK, PERK, p-eIF2α, eIF2α, ATF4, CHOP, and caspase 12 compared with the model group. The findings demonstrate that TRWE exerts a protective effect on kidney tissues in the presence of CP-induced inflammation.

## 4. Discussions

CP-induced nephrotoxicity is a common dose-limiting side effect in chemotherapy, primarily mediated by drug accumulation in renal tubular epithelial cells that triggers oxidative stress, inflammatory responses, and ERS. TRWE, rich in polyphenolic compounds and antioxidants, mitigates such damage by neutralizing free radicals and reducing inflammatory cytokines, thereby preserving renal tubular integrity and function. Critically, the multi-target nature of TRWE’s renoprotective mechanisms provides a direct rationale for employing NP. This field applies the principles of systems biology and a holistic perspective on network equilibrium to elucidate disease progression, which is characterized by its focus on multiple genes and targets [45]. From an NP perspective, it is a promising research area to comprehensively investigate the impact of *Taraxacum* root on kidney injury through the utilization of diverse database resources [46]. However, it is important to note that network pharmacology predictions are inherently hypothetical and require rigorous experimental validation. In this study, the effect of *Taraxacum* on kidney injury was studied by NP, and the target of *Taraxacum* on kidney injury was enriched by Gene Ontology (GO) enrichment analysis and Kyoto Encyclopedia of Genes and Genomes (KEGG) enrichment analysis. Furthermore, GMFA (GeneMANIA-based Functional Association) analysis enhanced NP predictions by investigating functional relationships among hub targets. Based on enrichment analysis of PERK-mediated unfolded protein response, the PERK/eIF2α/ATF4 signaling pathway was obtained through further screening. NP mapping revealed *Taraxacum’s* multi-target therapeutic network against renal injury through this mechanistic hierarchy, delineating its capacity to simultaneously modulate critical signaling cascades and pathological processes. This systems-level perspective not only elucidates the herb’s polypharmacological strategy but also provides a framework for validating its nephroprotective mechanisms in vivo while guiding exploration of *Taraxacum’s* understudied molecular targets in kidney injury pathophysiology.

This study aimed to explore the potential nephroprotective effects of TRWE against CP-induced AKI through in vivo experiments. Utilizing a CP-induced AKI murine model, we systematically evaluated the capacity of TRWE to mitigate renal impairment while preserving therapeutic outcomes. Currently, blood urea nitrogen (BUN) and serum CRE serve as common biomarkers for diagnosing drug-induced AKI [47]. Cisplatin-induced renal injury manifests as apoptotic and necrotic alterations in tubular epithelial cells alongside structural renal damage. Our findings suggest that TRWE ameliorates CP-induced elevations in BUN and CRE levels and restores renal histomorphology.

Prior studies have established the pivotal role of oxidative stress in CP-induced nephrotoxicity [48]. Upon cellular entry, CP generates ROS, with superoxide anion (O_2_) being a critical mediator of AKI [49]. Evaluation of these oxidative biomarkers suggests the therapeutic efficacy of TRWE in attenuating CP-induced renal injury, as indicated by reduced MDA accumulation and concomitant restoration of SOD/CAT activity, implying redox homeostasis modulation. Notably, the optimal TRWE dose identified here (400 mg/kg) diverges from prior neuropharmacological studies reporting bioactivity at 200 mg/kg in murine models [50]. This discrepancy may arise from differences in experimental models and drug administration sites, leading to distinct optimal dosages.

CP is recognized to induce aberrant protein accumulation or misfolding within the ER and ERS [51]. ERS elicits ER luminal disturbances, unfolded protein aggregation, and Ca^2+^ dysregulation, prompting the unfolded protein response (UPR) to restore cellular homeostasis [52]. During PERK activation, phosphorylated eIF2α facilitates ATF4 upregulation [53]. Network pharmacology predictions further suggest that TRWE might confer renal protection via modulation of ER stress and apoptosis pathways. These computational results provide a valuable starting point for hypothesis generation; however, their biological relevance remains speculative until experimentally tested. To explore TRWE’s potential in regulating tubular cell apoptosis through ER pathways, we assessed the expression of PERK, eIF2α, ATF4, CHOP, and caspase-12. Moderate ERS activates adaptive cytoprotective mechanisms [54]; however, excessive or prolonged ERS promotes apoptosis via ATF4-mediated induction of the pro-apoptotic factor CHOP [55]. Caspase-12, uniquely localized to the ER among caspases, is ubiquitously expressed across tissues. Our results indicate CP-induced upregulation of CHOP and a marked increase in cleaved caspase-12 expression. In this study, TRWE upregulated anti-apoptotic factors Bcl-2 and Bcl-XL while suppressing pro-apoptotic proteins Bax, Bad, and caspase-3 activation, thereby exerting anti-apoptotic effects. These observations align with reports by Fan et al., who identified PERK activation and CHOP-mediated renal epithelial apoptosis under ER stress [56]. In summary, this study integrated bioinformatics predictions with in vivo experimental validation to investigate the mechanistic basis of TRWE’s potential nephroprotective effects. The experimental results provided critical validation of the computational predictions, confirming the involvement of the PERK/eIF2α/ATF4 pathway and apoptosis regulation, thereby strengthening the reliability of the initial network pharmacology findings. However, several limitations should be considered when interpreting these findings. Firstly, although our network pharmacology analysis and preliminary experimental data suggest a multi-target mechanism, the precise molecular interactions and specific targets of the active components in TRWE require further validation through in vivo cellular experiments or loss-of-function/gain-of-function studies (e.g., knockdown or overexpression experiments). This step is essential to move beyond correlation and establish causal relationships between target modulation and biological effects. Secondly, as a natural product extract, the composition and potency of TRWE may be influenced by factors such as plant origin, harvest season, extraction methodology, and batch-to-batch variability, which could affect the reproducibility of our findings. In future studies, we will continue to use dandelion roots harvested in May from Jingyu and will also incorporate more standardized processing and detailed chemical characterization to ensure extract consistency and reliability. Additionally, the current study primarily focused on the PERK/eIF2α/ATF4/CHOP signaling axis, so other pathways potentially involved in TRWE’s renoprotective effects warrant further exploration. Despite these limitations, our study provides a foundational framework for understanding TRWE’s protective role against CP-induced nephrotoxicity. Coupled with its established anticancer properties, TRWE shows potential not only as a renoprotective adjunct to CP therapy but also as a synergistic complementary agent for oncological treatment.

## 5. Conclusions

This study adopted an integrated research paradigm combining network pharmacology and experimental validation to systematically elucidate the multi-target nephroprotective mechanisms of TRWE. Computational biology approaches identified 52 core targets closely associated with renal injury, preliminarily revealing TRWE’s therapeutic potential through regulation of apoptosis and ER stress signaling pathways. A protein–protein interaction network constructed via the GeneMANIA platform validated network pharmacology predictions, particularly emphasizing biological relevance within apoptotic pathways. Molecular docking simulations suggested potential binding affinities between TRWE’s primary active constituents (CGA, CA, CCA, and CTA) and key ER stress regulatory proteins (PERK, eIF2α, and ATF4). In vivo experiments further confirmed that TRWE markedly ameliorated cisplatin-induced renal oxidative damage, apoptosis, and dysfunction via modulation of the PERK/eIF2α/ATF4 signaling axis, thereby mechanistically elucidating the molecular basis of this traditional herb’s nephroprotection. Although these findings provide robust preclinical evidence, several challenges remain in translating these results into clinical applications. The complexity of multi-component interactions in herbal medicine necessitates further pharmacokinetic and pharmacodynamic studies to clarify bioavailability, metabolism, and potential synergistic effects in humans. Additionally, the extrapolation from cisplatin-induced rodent models to human chronic kidney diseases requires caution, and future validation in patient-derived samples or clinical trials is essential to confirm the relevance of the PERK/eIF2α/ATF4 pathway in human nephroprotection. This investigation provides the first systematic elucidation of *Taraxacum*’s modern pharmacological mechanisms from an ER stress perspective (Figure 10C) while acknowledging that the complexity of network regulation involving multi-component synergistic interactions in herbal medicine warrants further exploration.

## Figures and Tables

**Table 1:** Table of phytochemical constituents and their corresponding molecular targets.

Phytochemical	Target	Phytochemical	Target	Phytochemical	Target	Phytochemical	Target
Caffeic acid	MAPK1	Caftaric acid	MAPK1	Cichoric acid	MAPK1	Chlorogenic acid	ABCB1
	TLR4		MMP9		MMP9		MMP2
	MMP9		TTR		TTR		EDNRA
	TTR		EGFR		EGFR		ELANE
	EGFR		MMP2		MMP2		APP
	PIK3CA		MMP1		MMP1		SLC37A4
	STAT3		ELANE		APP		PRKCD
	NFE2L2		APP		ERBB2		PRKCA
	F3		ERBB2		PTGS1		AKR1B1
	MMP2		PTGS1		AKR1B1		MMP12
	MMP1		AKR1B1		ESR1		CA9
	ELANE		ESR1		MMP12		CA2
	MIF		MMP12		FYN		PDE4D
	APP		FYN		PYGL		MMP13
	ERBB2		MMP13		SLC6A2		BACE1
	CYP2C9		PYGL		AKR1C2		PDE5A
	CYP3A4		SLC6A2		LCK		PYGL
	PTGS1		AKR1C2		AKR1C4		CA1
	AKR1B1		LCK		AKR1B10		AKR1B10
	ESR1		AKR1C4				
	CA9		AKR1B10				
	CYP2C19						
	FYN						
	ALOX5						
	PIK3CB						
	PTPN1						
	CA2						
	SYK						
	CYP1A2						
	CA4						
	ESR2						
	HSD11B1						
	SLC6A2						
	AKR1C2						
	LCK						
	AKR1C4						
	CA3						
	CA1						
	CA6						
	AKR1B10						
	CA5A						

## Data Availability

The original contributions presented in this study are included in the article/supplementary material. Further inquiries can be directed to the corresponding authors.

## Supplementary Materials

The following supporting information can be downloaded at https://www.mdpi.com/article/10.3390/nu17193092/s1, Table S1. Drug targets and disease targets. Table S2. Phenolic compounds as AKI common targets. Table S3. Network pharmacology-informed functional annotation of TRWE’s nephroprotective mechanisms using GO/KEGG enrichment analyses. Table S4. GeneMANIA-based network expansion revealing 196 functional targets in *Taraxacum* nephroprotection. Table S5. Validation of the network pharmacology predictions using GO and KEGG enrichment analyses of the GeneMANIA-constructed functional gene network (FGN). Table S6. Molecular docking parameters and results of four phytochemicals in *Taraxacum* binding with PERK/ATF4/eIF2α.

## Abbreviations

CP	cisplatin
ER	endoplasmic reticulum
UPR	unfolded protein response
TRWE	water extract from *Taraxacum* roots
BUN	blood urea nitrogen
CRE	creatinine
MDA	malondialdehyde
SOD	superoxide dismutase
CAT	catalase
ERS	endoplasmic reticulum stress
PERK	protein kinase R-like ER kinase
eIF2α	eukaryotic initiation factor 2α
ATF4	activating transcription factor 4
CHOP	Children’s Hospital of Philadelphia
CTA	caftaric acid
CGA	chlorogenic acid
CA	caffeic acid
CCA	cichoric acid
AKI	acute kidney injury
ROS	reactive oxygen species
GO	Gene Ontology
KEGG	Kyoto Encyclopedia of Genes and Genomes
